# Interleukin-6 and lipoprotein-associated phospholipase A2 are associated with functional trajectories

**DOI:** 10.1371/journal.pone.0214784

**Published:** 2019-04-01

**Authors:** Mandip S. Dhamoon, Ying-Kuen Cheung, Yeseon P. Moon, Clinton B. Wright, Ralph L. Sacco, Mitchell S. V. Elkind

**Affiliations:** 1 Department of Neurology, Icahn School of Medicine at Mount Sinai, New York, NY, United States of America; 2 Department of Epidemiology, Mailman School of Public Health, Columbia University, New York, NY, United States of America; 3 Department of Biostatistics, Mailman School of Public Health, Columbia University, New York, NY, United States of America; 4 Department of Neurology, College of Physicians and Surgeons, Columbia University, New York, NY, United States of America; 5 National Institutes of Health, Bethesda, MD, United States of America; 6 McKnight Brain Institute, Miller School of Medicine, University of Miami, Miami, FL, United States of America; 7 Departments of Neurology, Miller School of Medicine, University of Miami, Miami, FL, United States of America; 8 Public Health Sciences, Miller School of Medicine, University of Miami, Miami, FL, United States of America; 9 Human Genetics, Miller School of Medicine, University of Miami, Miami, FL, United States of America; Cardiff University, UNITED KINGDOM

## Abstract

**Background/Objectives:**

Inflammatory biomarkers have been associated with stroke and mortality, but inflammation may also have detrimental effects beyond acute events. We hypothesized that serum concentrations of interleukin-6 (IL6) and lipoprotein-associated phospholipase A2 (LpPLA2) were inversely associated with long-term functional decline independently of vascular risk factors, stroke and myocardial infarction (MI) occurring during follow-up.

**Design:**

Prospective population based cohort study

**Setting:**

The Northern Manhattan Study

**Participants (including the sample size):**

Race/ethnically diverse stroke-free individuals in northern Manhattan aged ≥40 years (n = 3298).

**Intervention:**

None

**Measurements:**

Annual functional assessments with the Barthel index (BI), for a median of 13 years. BI was analyzed as a continuous variable (range 0–100). Baseline demographics, risk factors, and laboratory studies were collected, including IL6 (n = 1679), LpPLA2 mass (n = 1912) and activity (n = 1937). Separate mixed models estimated standardized associations between each biomarker and baseline functional status and change over time, adjusting for demographics, vascular risk factors, social variables, cognition, and depression measured at baseline, and stroke and MI occurring during follow-up.

**Results:**

Mean age was 69 (SD 10) years, 35% were male, 53% Hispanic, 74% hypertensive, and 16–24% diabetic. LogIL6 was associated with decline in BI over time (-0.13 points per year, 95% CI -0.24, -0.02) and marginally with baseline BI (-0.20, 95% CI -0.40, 0.01). LpPLA2 activity levels were associated with baseline BI (-0.36, 95% CI -0.68, -0.04) but not change over time, and LpPLA2 mass levels were not associated with either.

**Conclusion:**

In this large population-based study, higher serum inflammatory biomarker levels were associated with disability, even when adjusting for baseline covariates and stroke and MI occurring during follow-up.

## Introduction

Inflammatory pathways have been implicated in various disease processes, including vascular and cerebrovascular disease, neurodegenerative conditions, and arthritis. Interleukin-6 (IL6) is an inflammatory cytokine that stimulates secretion of C-reactive protein systemically, but is also elevated locally with brain injury, predominantly expressed in brain white matter, and may stimulate growth factors.[1] Elevated IL6 has been associated with various dementias and stroke. Lipoprotein-associated phospholipase-A2 (LpPLA2) is secreted by immune cells in arterial walls and is involved in inflammatory processes in atherosclerotic plaque.[2] Elevated LpPLA2 has been associated with coronary heart disease and vascular events.[3] However, the association between these biomarkers and long-term functional trajectories is unclear, and these biomarkers may allow the prognostication not only of vascular events but also of functional trajectories, as we have found in previous research.[4]

We hypothesized that elevated serum levels of IL6 and LpPLA2 are independently associated with worse functional trajectories in those free of stroke at baseline, and there are several unique approaches of this analysis. We estimated 2 components of functional trajectories, baseline function and change over time. Estimating trajectories may reveal courses of change and specific predictors that are not captured with crude analysis of change over 2 time points. Also, we analyzed the components of function using subdomains of a functional scale.

## Methods

The Northern Manhattan Study (NOMAS) population-based prospective cohort of those free of stroke at baseline included 3298 participants recruited by random digit dialing of published and unpublished telephone numbers from 1993–2001. Participants were enrolled if they: 1) were ≥40 years of age; 2) lived in a pre-defined geographic area of northern Manhattan for ≥3 months in a household with a telephone; and 3) had no history of stroke. The study was approved by IRBs of Columbia University and University of Miami, and informed consent was obtained from all participants. Further characteristics of the cohort have been published.[5–7]

### Baseline evaluation

Bilingual research assistants collected data using standardized questions regarding the following conditions, as previously described: hypertension, diabetes mellitus, hypercholesterolemia, cigarette smoking, alcohol use, and cardiac conditions.[8] Participants underwent a comprehensive medical history, neurological examination, review of medical records, functional status assessed by the Barthel index (BI), quality of life (QOL) assessed by the Spitzer QOL index (QLI), and fasting blood samples.

### Follow-up

Participants were followed annually via phone screening to detect change in vital status, new neurological or cardiac symptoms and events, interval hospitalizations, cognitive function, and functional status via the BI. Cause and date of death were determined from multiple sources, including proxy report, medical record review, and review of death certificates when available. Only two subjects were completely lost to follow-up after their baseline examination, and the average annual contact rate was 99%.

A positive screen for any potential cardiac or neurological event was followed by an in-person assessment to determine whether a vascular outcome occurred. In addition, all admissions and discharges were screened for hospitalizations and outcomes that may not have been captured by telephone interview. Hospital records were reviewed to classify outcomes as previously reported.[7] Stroke included ischemic stroke, intracerebral hemorrhage, and subarachnoid hemorrhage. At least 2 stroke neurologists verified and classified all stroke cases. MI required ≥2 of the 3 following criteria: (a) ischemic cardiac pain determined to be typical angina; (b) cardiac marker abnormalities (abnormal CK-MB fraction or troponin I values); and (c) ischemic EKG abnormalities. MI diagnosis was adjudicated by cardiologists independently after review of all clinical data, and definite and probable MI were included.

### Study outcome

The BI[9,10] measures performance in 10 activities of daily living (ADLs) and ranges from 0–100 in 5-point increments, with 100 indicating normal physical functioning. Previous research has demonstrated the reliability of phone functional assessments using the BI.[11] Although it is an ordinal scale, the BI may be analyzed as a continuous variable for increased power to detect associations, ability to describe the course of change over time in linear form, and avoidance of potential misclassification due to crude categorization.[12–14] We refer to “functional status” in this manuscript to denote the use of a functional scale, where each individual item is a score of functional ability as opposed to a score of disability. The BI is not a direct measure of physical performance (such as grip strength or gait speed), but a self- or proxy-assessment of ability to perform certain ADL tasks.

### Explanatory variables

Laboratory personnel were blinded to patient clinical data and markers were run in the same participants. Serum samples for IL6 were drawn into EDTA tubes at baseline, spun immediately at 3,000 g at 4°C for 20 min, and frozen at –70°C for later analysis. IL6 concentrations were then measured in batched samples by enzyme-linked immunosorbent assay using monoclonal antibodies to IL6 with a lower limit of detection of 0.1 pg/mL (Biosource International, Camarillo, Calif., USA). For LpPLA2 mass and activity levels, samples were thawed to 4°C and analyzed within one hour. Serum samples were assayed for levels of LpPLA2 mass, using a manual enzyme-linked immunoassay (PLAC), and LpPLA2 activity, using a colorimetric activity method (CAM), at a central laboratory at diaDexus, Inc., San Francisco, CA, USA. Quality control was maintained using standardized procedures including running samples in duplicate. The above assays were run only among a sub-sample of the entire NOMAS cohort because available funding only allowed this amount of assays to be run.

Biomarker measurement distributions (IL6, LpPLA2 mass assay, and LpPLA2 activity assay) were determined. Since IL6 was right-skewed, log transformation was performed for the main analysis. Although not required to satisfy model assumptions, log transformations approximated a normal distribution of the variable to be consistent with prior analyses. LpPLA2 mass and activity values were centered to the mean of all values.

### Covariates

Analytic models were adjusted for the following: demographic variables (age, sex, race-ethnicity), medical risk factors (body mass index [body weight in kilograms divided by the square of height in meters], hypercholesterolemia [defined by self-report, lipid lowering therapy use, or fasting total cholesterol level >240 mg/dL], diabetes mellitus [defined by self-report, fasting blood glucose level ≥126 mg/dL, or insulin/oral hypoglycemic use], hypertension [defined as a systolic blood pressure recording ≥140 mmHg or a diastolic blood pressure recording ≥90 mm Hg based on the average of two blood pressure measurements or the participant’s self-report of a history of hypertension or antihypertensive use]), smoking (defined as either nonsmoker or smoker within the last year), alcohol use (with moderate alcohol use classified as 1 drink/month to 2 drinks/day), any physical activity (versus none), social variables (marital status, insurance status [classified as uninsured/Medicaid versus Medicare/private insurance], number of friends [individuals whom the participant knows well enough to visit in their homes], years living in the community), and cognitive/mood factors (depression [defined as a Hamilton Depression scale score of ≥12], performance on mini-mental state examination [analyzed as a continuous variable], and Spitzer quality of life scale score).[15]

### Statistical analysis

We sought to determine whether levels of inflammatory biomarkers were associated with baseline BI and a steeper slope of decline over time. We compared variable distributions based on availability of inflammatory lab data. We compared time trends of BI in the cohort with measurements to that without measurements using an interaction term between availability of measurements and time, in unadjusted and fully adjusted models.

We tested each biomarker in separate linear mixed models. We assessed the association between inflammatory biomarker levels and repeated measurements of BI, adjusting sequentially for: baseline demographic variables, medical risk factors, smoking and alcohol use and physical activity, social variables, and cognitive/mood factors, as defined above. In order to assess whether levels were associated with change in outcomes over time, we included interaction terms between time of follow-up assessment and the biomarker. Model diagnostics including tests of linearity and goodness of fit measures (comparing nested models via likelihood ratio tests) were used to evaluate the final model. Spline regression was performed to evaluate lack of linearity in the association between inflammatory biomarker levels and functional trajectories. There was no evidence to suggest lack of linearity in the final models. In order to assess whether interval vascular events such as stroke and MI were implicated in the trajectory of functional status, we also adjusted for stroke and MI as time-varying covariates.

We also examined whether the relationship between inflammatory biomarker levels and functional status differed for mobility (including the BI tasks of: transfers, mobility, and stair use) and non-mobility (including the BI tasks of: feeding, bathing, grooming, dressing, bowels, bladder, and toilet use) domains of the BI, which we analyzed separately in unadjusted and fully adjusted models. Mobility and non-mobility domains were analyzed as separate outcomes in distinct models.

We examined the effect of hospitalization on functional status, and determined whether loss to follow-up was related to hospitalization, which could have introduced bias in the estimation of functional trajectories. Specifically, in cases of hospitalization during follow-up, we assessed the time between pre-hospitalization BI assessment and post-hospitalization BI assessment, comparing non-stroke/MI hospitalization to all hospitalizations. We also examined the impact of loss to follow-up by calculating the time between last functional assessment and death and examining the distribution of the last ADL score measured among those who died. We also examined records for individuals who had maximum follow-up time <10 years to identify possible reasons for loss to follow-up.

## Results

Table 1 shows distributions of variables stratified by availability of inflammatory labs. Table 2 shows summary statistics in each cohort. There were significant differences in the distributions of few variables based on availability of inflammatory lab data. The most relevant differences were lower prevalence of diabetes among those with IL6 measurements (p<0.0001), younger age among those with LpPLA2 mass and activity levels (p<0.0001), and higher prevalence of Medicaid or no insurance and diabetes among those with LpPLA2 mass and activity levels (p<0.0001 and p = 0.0001 respectively). The S1 Table lists the distribution of BI assessments over follow-up. The mean follow-up time was 11.5 years in those with IL6 measurements available, 11.4 years in those with LpPLA2 mass measurements available, and 11.5 years in those with LpPLA2 activity measurements available.

**Table 1:** Baseline characteristics of the cohort, by availability of inflammatory labs*.

Variable	IL6 available	LpPLA2 activity available	LpPLA2 mass available
**Number of participants, No. (%)**	1679 (100)	1912 (100)	1937 (100)
**Demographics:**			
Age, mean (SD), y	69.5 (10.3)	69.1 (10.2)	69.0 (10.2)
Male, No. (%)	594 (35.4)	677 (35.4)	689 (35.6)
Race-ethnicity:			
Non-Hispanic white, No. (%)	352 (21.0)	382 (20.0)	385 (19.9)
Non-Hispanic black, No. (%)	394 (23.5)	420 (22.0)	428 (22.1)
Hispanic, No. (%)	883 (52.6)	1056 (55.2)	1070 (55.2)
Other, No. (%)	50 (3.0)	54 (2.8)	54 (2.8)
Received at least high school education, No. (%)	780 (46.5)	842 (44.1)	851 (44.0)
Marital status, No. (%) married	528 (31.5)	604 (31.6)	615 (31.8)
Health insurance, No. (%)			
Medicaid or no insurance	754 (45.3)	898 (47.3)	912 (47.4)
Medicare or private insurance	912 (54.7)	1000 (52.7)	1011 (52.6)
**Vascular risk factors, No. (%)**			
Hypertension	1231 (73.3)	1420 (74.3)	1441 (74.4)
Diabetes mellitus	261 (15.6)	459 (24.1)	465 (24.1)
Hypercholesterolemia	1067 (63.6)	1207 (63.1)	1222 (63.1)
History of atrial fibrillation	81 (4.8)	88 (4.6)	87 (4.5)
History of coronary heart disease	359 (21.4)	422 (22.1)	426 (22.0)
Body mass index, mean (SD), kg/m^2^	27.8 (5.5)	28.0 (5.5)	28.1 (5.5)
Alcohol consumption:			
Never Drank	411 (24.5)	440 (23.0)	448 (23.1)
Past Drinker	389 (23.2)	483 (25.3)	488 (25.2)
Light Drinker	218 (13.0)	253 (13.2)	256 (13.2)
Moderate Drinker	574 (34.2)	636 (33.3)	644 (33.3)
Intermediate Drinker	59 (3.5)	69 (3.6)	69 (3.6)
Heavy Drinker	28 (1.7)	31 (1.6)	32 (1.7)
Physical activity, any	935 (55.7)	1049 (54.9)	1064 (54.9)
Smoking:			
Never	802 (47.8)	908 (47.5)	918 (47.4)
Former	629 (37.5)	698 (36.5)	709 (36.6)
Current	246 (14.7)	304 (15.9)	308 (15.9)
**Other medical conditions, No. (%)**			
Hamilton depression scale score, mean (SD)	3.3 (3.9)	3.3 (3.9)	3.3 (3.9)
Hamilton depression score ≥12	71 (4.3)	86 (4.6)	86 (4.5)
Mini mental state score, mean (SD)	25.9 (3.8)	25.9 (3.8)	25.9 (3.8)
Spitzer quality of life index score, mean (SD)	9.1 (1.3)	9.1 (1.3)	9.1 (1.3)

*IL6 = interleukin-6; LpPLA2 = lipoprotein phospholipase A2

**Table 2:** Summary statistics for each measurement cohort.

Characteristic	IL6 measurements available	LpPLA2 mass measurements available	LpPLA2 activity measurements available
Mean biomarker levels (SD)	109.7 pg/mL (723.8)	117.0 ng/mL (29.6)	308.7 nmol/min/mL (88.3)
Median biomarker levels (IQR)	1.56 pg/mL (0.80–2.88)	115.5 ng/mL (96.5–135.9)	306.7 nmol/min/mL (245.3–365.5)
Coefficient of variation	6.60	0.25	0.29
Number of first strokes occurring during follow-up	186	206	210
Number of first MI occurring during follow-up	130	136	137
Total number of BI assessments	19561	22470	22129
Mean BI assessments (median) per individual	11.7 (13)	11.6 (13)	11.6 (13)

IL6 = interleukin-6; LpPLA2 = lipoprotein phosopholipase-A2; SD = standard deviation; IQR = interquartile range; MI = myocardial infarction; BI = Barthel index

There were no differences in the time trend of BI when the sample with IL6 measurements was compared to the sample without IL6 measurements, in a fully adjusted model (p = 0.15), or with a similar comparison with LpPLA2 activity measurements (p = 0.8) and LpPLA2 mass measurements (p = 0.6).

Table 3 shows unadjusted and adjusted mixed models testing the association between logIL6 levels and trajectories of functional status. In an unadjusted model, there was an annual decline in BI score of 1.49 points per year, and an additional decline of 0.15 points per year per unit increase in logIL6. Also, with a unit increase in logIL6, there was a decrease in baseline BI score of 0.49 points. In a fully adjusted model, greater logIL6 levels were significantly associated with accelerated decline in function over time (-0.13 points per year, 95% CI -0.24, -0.02), and there was a trend for lower baseline functional status (-0.20, 95% CI -0.40, 0.01). In adjusted models (Table 4), higher LpPLA2 activity levels were associated with lower baseline BI score (-0.36, 95% CI -0.68, -0.04) but not with change of BI over time. LpPLA2 mass levels were not associated with either overall mean BI or change in BI over time, in unadjusted for fully adjusted models (Table 4).

**Table 3:** Associations between interleukin-6 levels and trajectories of functional status.

	Log of IL6 levels		
Variable	Change in BI score	95% CI	p-value
**Unadjusted model:**			
Annual change in BI score	-1.49	-1.62, -1.35	<0.0001
Change in BI score per unit increase in logIL6	-0.49	-0.79, -0.19	0.001
Additional annual change in BI score per unit increase in logIL6	-0.15	-0.24, -0.06	0.001
Adjusted for demographics:*			
Annual change in BI score	-1.43	-1.57, -1.30	<0.0001
Change in BI score per unit increase in logIL6	-0.39	-0.68, -0.09	0.01
Additional annual change in BI score per unit increase in logIL6	-0.13	-0.22, -0.05	0.003
Adjusted for vascular risk factors:**			
Annual change in BI score	-1.43	-1.57, -1.30	<0.0001
Change in BI score per unit increase in logIL6	-0.27	-0.56, 0.02	0.065
Additional annual change in BI score per unit increase in logIL6	-0.13	-2.9, -0.04	0.004
**Adjusted for mood and cognitive variables:**‡			
Annual change in BI score	-0.54	-0.73, -0.34	<0.0001
Change in BI score per unit increase in logIL6	-0.17	-0.38, 0.04	0.1
Additional annual change in BI score per unit increase in logIL6	-0.07	-0.18, 0.04	0.2
**Adjusted for stroke and MI:π**			
Annual change in BI score	-0.34	-0.53, -0.15	0.0006
Change in BI score per unit increase in logIL6	-0.20	-0.40, 0.01	0.066
Additional annual change in BI score per unit increase in logIL6	-0.13	-0.24, -0.02	0.02

IL6 = interleukin-6; BI = Barthel index; MI = myocardial infarction

*adjusted for: baseline age, sex, and race-ethnicity

**additionally adjusted for: diabetes, hypertension, coronary artery disease, hypercholesterolemia, physical activity, alcohol use, smoking, and body mass index

‡additionally adjusted for: marital status, insurance, number of friends, and years lived in the neighborhood, depression, mini-mental state score, and Spitzer quality of life index πadditionally adjusted for stroke or myocardial infarction occurring during follow-up

**Table 4:** Associations between lipoprotein-associated phospholipase-A2 levels and trajectories of functional status.

	LpPLA2 activity levels			LpPLA2 mass levels		
Variable	Change in BI score	95% CI	p-value	Change in BI score	95% CI	p-value
**Unadjusted model:**						
Annual change in BI score	-1.59	-1.71, -1.47	<0.0001	-1.59	-1.70, -1.47	<0.0001
Change in BI score per unit increase in biomarker	0.27	-0.15, 0.69	0.2	0.37	-0.04, 0.79	0.08
Additional annual change in BI score per unit increase	-0.04	-0.16, 0.08	0.5	-0.08	-0.19, 0.04	0.2
Adjusted for demographics:*						
Annual change in BI score	-1.52	-1.64, -1.40	<0.0001	-1.52	-1.63, -1.40	<0.0001
Change in BI score per unit increase in biomarker	0.23	-0.22, 0.68	0.3	-0.30	-0.14, 0.73	0.2
Additional annual change in BI score per unit increase	-0.04	-0.16, 0.08	0.5	-0.08	-0.20	0.03
Adjusted for vascular risk factors:**						
Annual change in BI score	-1.52	-1.63, -1.40	<0.0001	-1.51	-1.63, -1.40	<0.0001
Change in BI score per unit increase in biomarker	0.17	-0.27, 0.62	0.4	0.28	-0.14, 0.70	0.2
Additional annual change in BI score per unit increase	-0.04	-0.16, 0.08	0.5	-0.08	-0.20, 0.04	0.2
**Adjusted for mood and cognitive variables:**‡						
Annual change in BI score	-0.63	-0.79, -0.46	<0.0001	-0.61	-0.78, -0.45	<0.0001
Change in BI score per unit increase in biomarker	-0.35	-0.68, -0.03	0.03	0.07	-0.24, 0.38	0.6
Additional annual change in BI score per unit increase	0.12	-0.04, 0.29	0.1	0.003	-0.16, 0.16	0.9
**Adjusted for stroke and MI:π**						
Annual change in BI score	-0.47	-0.63, -0.31	<0.0001	-0.46	-0.62, -0.30	<0.0001
Change in BI score per unit increase in biomarker	-0.36	-0.68, -0.04	0.03	0.05	-0.26, 0.36	0.8
Additional annual change in BI score per unit increase	0.11	-0.05, 0.26	0.2	-0.01	-0.16, 0.14	0.9

LpPLA2 = lipoprotein phospholipase-A2; BI = Barthel index; MI = myocardial infarction

*adjusted for: baseline age, sex, and race-ethnicity

**additionally adjusted for: diabetes, hypertension, coronary artery disease, hypercholesterolemia, physical activity, alcohol use, smoking, and body mass index

‡additionally adjusted for: marital status, insurance, number of friends, and years lived in the neighborhood, depression, mini-mental state score, and Spitzer quality of life index πadditionally adjusted for stroke or myocardial infarction occurring during follow-up

For the sensitivity analysis of BI domain as well as three other sensitivity analyses, models did not significantly differ from the primary analyses and are not presented here: 1) including possible MI in the definition of MI; 2) excluding those with baseline coronary artery disease; and 3) excluding those with baseline BI<100 (n = 422).

The relationship between hospitalization and functional status was assessed to identify potential bias related to loss to follow-up. For a non-stroke/MI hospitalization, the amount of time between pre-hospitalization assessment and post-hospitalization assessment was on average 1.09 years, with an upper quartile of only 1.11 years. For all hospitalizations, the corresponding interval was on average 1.05 years, with an upper quartile of 1.11 years. These results suggest that hospitalization did not cause selective loss to follow-up or introduce bias into the timing of BI assessments, which were scheduled to occur annually.

The relationship between last follow-up and death was also assessed. The average time between last functional assessment and death was 0.74 years, with an upper quartile of 0.93 years. Those with last BI score before death of <60 more often had a shorter interval between last functional assessment and death (61.2% with interval <0.5 years) compared to those with BI of 60–90 (37.0%) or 95–100 (33.8%). When we examined individual records for those with maximum follow-up time <10 years, we found no systematic reasons for loss to follow-up.

## Discussion

We found that inflammatory biomarker levels were independently associated with trajectories of functional status over the long term in a large, population-based study with frequent, regular measurements of functional status. There was an overall functional decline in the entire cohort, and increasing levels of logIL6 were associated with additional annual decline over time. LpPLA2 activity levels were associated with lower baseline functional scores but not change over time. These changes were independent of stroke and MI, since we adjusted for the occurrence of these events during follow-up, and were seen in both mobility and non-mobility domains of the BI, suggesting a general impact of these biomarker levels on functional status that may not be mediated only through pathways controlling movement and motion. Furthermore, there was limited loss to follow-up from hospitalization or death.

Several previous studies have examined outcomes related to IL6 concentrations among population-based cohorts. In a prior analysis in NOMAS among 1224 participants,[16] IL6 concentrations above the median were associated with greater decline in cognitive ability measured by the modified Telephone Interview for Cognitive Status over a median of 3 years of follow-up. In a cross-sectional meta-analysis of 6 cohorts, concentrations of circulating biomarkers were tested for associations with measures of physical performance.[17] Higher levels of five inflammatory markers, including IL6, were associated with worse physical performance. Among 2979 individuals aged 70–79 years,[18] there was a greater risk of incident mobility limitation with higher IL6 (RR 1.19, 95% CI 1.10–2.8) over 30 months of follow-up. In another analysis in this cohort,[19] 2234 elderly individuals were followed for a median of 11.4 years, and higher IL6 concentrations were associated with the onset of disability and mortality. Higher IL6 concentrations have also been independently associated with periventricular and deep white matter hyperintensity volume (WMHV) among 137 elderly women, suggesting a possible mediating effect between inflammatory states and disability.[20]

The associations between IL6 and outcomes have also been examined in particular subgroups. For example, in a meta-analysis of 4112 stroke patients from 20 studies,[21] IL6 was associated with poor outcome, defined as MRS score of >2 or BI score of <85, as well as post-stroke infection. Among 80 individuals with vascular dementia, greater IL6 concentrations were independently associated with lower BI scores in a cross-sectional analysis.[22] Among 1727 individuals >70 years of age in the Duke Established Populations for Epidemiologic Studies of the Elderly study,[23] higher IL6 concentrations were associated with disability and self-rated health, and IL6 concentrations were positively associated with cancer, heart attack, and hypertension. Among 3925 men aged 60–79 years, higher IL6 concentrations were associated with incident mobility limitation over an average of 11.5 years of follow-up.[24] The current analysis extends these previous findings by demonstrating an association between IL6 levels and accelerated decline in functional status over time, independent of the occurrence of vascular events.

Although associations between LpPLA2 and functional status have not been rigorously studied previously, to our knowledge, LpPLA2 has been associated with vascular outcomes in previous studies. In a prior NOMAS analysis among 467 individuals with first ischemic stroke, LpPLA2 was independently associated with recurrent stroke (HR 2.08, 95% CI 1.04–4.18) and the composite outcome of recurrent stroke, MI, or vascular death.[25] In another NOMAS analysis of 1946 stroke-free participants,[26] LpPLA2 mass levels were associated with incident large-artery ischemic stroke among non-Hispanic Whites. In another NOMAS analysis,[27] LpPLA2 levels were associated with white matter hyperintensity volume. In a large collaborative study using data from 32 prospective studies and involving 79036 patients,[3] LpPLA2 mass and activity were independently associated with increased risk of vascular events, including MI and stroke, and vascular mortality. In the Clopidogrel in High-Risk Patients with Acute Non-disabling Cerebrovascular Events trial,[28] higher baseline LpPLA2 activity levels were independently associated with higher risk of 90-day stroke as well as a composite of ischemic stroke, myocardial infarction, or death. Expert panels have recommended measuring LpPLA2 to improve risk prediction of cardiovascular disease,[29] and the current analyses suggest that it may also be effective to predict baseline functional status. We did not observe an association between LpPLA2 mass levels and baseline functional status or change over time. This lack of association may be because activity levels may more accurately reflect the impact of the LpPLA2 enzyme on functional status, compared to LpPLA2 mass.

There are several mechanisms by which IL6 may be associated with accelerated functional decline over time. IL6 triggers chronic elevations of C-reactive protein and has been elevated in elderly populations possibly as a result of increased adiposity, hormonal changes associated with aging, and physiological stress.[30] Inflammation has been implicated in atherogenesis, which may lead to the accumulation of clinical and subclinical events that negatively impact functional status. Also, dementia pathogenesis is known to involve inflammatory processes, in which IL6 has been implicated.[31] Indeed, inflammatory processes may be the primary drivers of the structural and functional brain changes seen in the disease, and not just responses to abnormal buildup of proteins.[32] Elevated IL6 concentrations may reflect early stages of neurodegenerative conditions that may cause disability. We also found that LpLA2 activity levels were associated with lower baseline functional status but not with change over time. Considering its strong association with vascular events, elevated LpPLA2 enzymatic activity may be a marker of prior subclinical vascular injury that would negatively affect baseline functional status estimated in this study. A growing body of research demonstrates that particular interventions, targeted to inflammatory pathways, may reduce the incidence of vascular events. For example, canakinumab, a therapeutic monoclonal antibody targeting interleukin-1β, has been shown to be effective to reduce the rate of recurrent cardiovascular events.[33] The present research would inform the extension of such treatments to target functional status and disability, in addition to vascular events.

Another potential mechanism linking inflammatory markers and accelerated functional decline may involve loss of muscle mass. Systemic inflammatory processes may trigger a catabolic response in the body, including muscle wasting and sarcopenia[34], which would in turn affect ability to perform ADLs. IL6 in particular has been associated with loss of muscle mass in animal and human studies, with either direct or indirect effects on atrophy.[35]

This study has several strengths. There was no evidence of bias related to hospitalization on data ascertainment and no evidence of any effect of hospitalization on timing or regularity of follow-up assessments. Also, there was no evidence that there was loss to follow-up in the last functional assessments before death, and overall the average length of follow-up among survivors was long (11 years). This study was in a large, population-based cohort with repeated, regular measurements of functional status with a validated scale. There was also regular surveillance for vascular events and hospitalization and expert adjudication of events. Biomarkers were measured according to standard procedures, and confounders were adjusted for in models. Among studies of inflammatory biomarkers, we were unable to find others in which both baseline functional status as well as the trajectory of change over time was analyzed, not only disability measured at a single time point. Furthermore, this study is among the first to describe associations between LpPLA2 and functional outcomes.

One limitation of the proposed study involves deficiencies in the primary outcome measure. The BI is subject to ceiling effects and is insensitive to small changes in disability.[36] Analyzing the BI as a continuous variable is advantageous since this approach can capture and quantify the variance and course of change over time, which would likely not be captured by using a categorical or dichotomous variable.[13,14] Also, we focused on a self-reported measure of ADLs as opposed to performance-based measures of functioning, which may have captured alternate aspects of functional status. Finally, NOMAS is a sample of an urban, predominantly Hispanic population, and findings may not be generalizable to other settings or population demographics.

Further research on inflammatory biomarkers is needed that tests associations with trajectories of functional status, not just single functional measurements in time. Also, research is needed that tests potential interventions to mitigate functional decline. The first step is to identify those at risk of deterioration. Then, several potential approaches may be effective that target inflammatory pathways. For example, statins and other cholesterol-modifying agents reduce levels of LpPLA2[29] and may play a role in reducing inflammation-mediated declines in function. Medications specifically targeted to the mechanism of biomarkers, such as darapladib to inhibit the activity of LpPLA2, may also be effective.[29]

## Supporting information

S1 Table Distribution of Barthel index assessments over follow-up. (DOCX)
