# Supplementary material for: Interleukin-6 and lipoprotein-associated phospholipase A2 are associated with functional trajectories
Source: PLoS One. 2019 Apr 1;14(4):e0214784. doi: 10.1371/journal.pone.0214784 (PMC6443177; doi:10.1371/journal.pone.0214784)
Supplement: S1 Table — (DOCX) [file pone.0214784.s001.docx]

**Supplementary Table**: Distribution of Barthel index assessments over follow-up

|  | **Number of Barthel index assessments** | | |
| --- | --- | --- | --- |
| **Time period of follow-up (years)*** | **IL6 measurements available** | **LpPLA2 mass measurements available** | **LpPLA2 activity measurements available** |
| 1 | 1360 | 1594 | 1610 |
| 2 | 1423 | 1657 | 1673 |
| 3 | 1479 | 1622 | 1643 |
| 4 | 1416 | 1600 | 1622 |
| 5 | 1364 | 1543 | 1566 |
| 6 | 1260 | 1441 | 1463 |
| 7 | 1264 | 1430 | 1455 |
| 8 | 1237 | 1386 | 1408 |
| 9 | 1158 | 1305 | 1328 |
| 10 | 1136 | 1267 | 1290 |
| 11 | 1068 | 1216 | 1237 |
| 12 | 1058 | 1186 | 1206 |
| 13 | 891 | 998 | 1019 |
| 14 | 697 | 827 | 834 |
| 15 | 461 | 544 | 553 |
| 16 | 265 | 263 | 269 |
| 17 | 142 | 131 | 138 |
| 18 | 95 | 86 | 89 |
| 19 | 80 | 72 | 78 |
| 20 | 25 | 40 | 41 |
| 21 | 4 | 5 | 7 |

*the time period incorporates the ½ year before and the ½ year after the time interval. So for the time period of 1 year, the time interval is 0.5 <= time < 1 year.
